# In search of ecological determinants of fungal infections: A semi‐field experiment with folivorous moths

**DOI:** 10.1002/ece3.8926

**Published:** 2022-05-24

**Authors:** Robin Gielen, Kadri Põldmaa, Toomas Tammaru

**Affiliations:** ^1^ 37546 Entomology Unit Department of Zoology Institute of Ecology and Earth Sciences Faculty of Science and Technology University of Tartu Tartu Estonia; ^2^ 37546 Mycology Unit Department of Botany Faculty of Science and Technology Institute of Ecology and Earth Sciences University of Tartu Tartu Estonia; ^3^ 37546 Natural History Museum and Botanical Garden University of Tartu Tartu Estonia

**Keywords:** endophyte, entomopathogen, Hypocreales, life history, mortality, prevalence

## Abstract

Natural enemies shape the fate of species at both ecological and evolutionary time scales. While the effects of predators, parasitoids, and viruses on insects are well documented, much less is known about the ecological and evolutionary role of entomopathogenic fungi. In particular, it is unclear to which extent may the spatiotemporal distribution patterns of these pathogens create selective pressures on ecological traits of herbivorous insects. In the present study, we reared three lepidopteran species in semi‐natural conditions in a European hemiboreal forest habitat. We studied the probability of the insects to die from fungal infection as a function of insect species, food plant, study site, (manipulated) condition of the larvae, and the phenological phase. The prevalence of entomopathogenic fungi remained low to moderate with the value consistently below 10% across the subsets of the data while as many as 23 fungal species, primarily belonging to the families Cordycipitaceae, Aspergillaceae, and Nectriaceae, were recorded. There were no major differences among the insect species in prevalence of the infections or in the structure of associated fungal assemblages. The family Cordycipitaceae, comprising mainly obligatory entomopathogens, dominated among the pathogens of pupae but not among the pathogens of larvae. Overall, there was evidence for a relatively weak impact of the studied ecological factors on the probability to be infected by a fungal pathogen; there were no effects of food plant, study site, or phenology which would be consistent over the study species and developmental stages of the insects. Nevertheless, when the prevalence of particular fungal taxa was studied, *Akanthomyces muscarius* was found infecting insects fed with leaves of only one of the food plant, *Betula* spp. Feeding on a particular plant taxon can thus have specific fitness costs. This demonstrates that fungus‐mediated effects on insect life history traits are possible and deserve attention.

## Graphical abstract

The probability of the insects to die from fungal infection was studied as dependent on insect species, food plant of the insect, study site, (manipulated) condition of the larvae, and the phenological phase. In the course of rearing, visually detectable fungal infections were isolated and identified. The prevalence of entomopathogenic fungi remained moderate with the value consistently below 10% across the subsets of the data while as many as 23 fungal species, primarily belonging to the families Cordycipitaceae, Aspergillaceae, and Nectriaceae, were recorded. Overall, there was evidence for a relatively weak impact of ecological factors on the probability to be infected by a fungal pathogen; there were no effects of host plant, study site, or phenology which would be consistent over the study species and developmental stages of the insects.

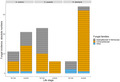

## 1 INTRODUCTION

For most organisms, natural enemies constitute an environmental factor of major ecological significance. In the case of insects, in general, most juveniles succumb to predators and parasitoids before the adult stage is attained (Cornell & Hawkins, 1995; Peterson et al., 2009). As a consequence, these natural enemies are considered key determinants of insect population dynamics (Price et al., 2011). From another perspective, spatial and temporal differences in predation pressure have the potential to create significant selective pressures on insect life histories. In particular, seasonal differences in mortality imposed by insectivorous birds (Remmel et al., 2011) are among the most important determinants of species‐specific phenologies (Tammaru et al., 2001), and may also well be responsible for the frequently observed among‐generation differences in insect body size (Remmel et al., 2011; Tedersoo et al., 2020). Furthermore, predation pressures specific to plant species may constitute a selective factor shaping food plant use of herbivorous insects (Murphy & Loewy, 2015). As pathogens may cause comparable or even higher mortality among their insect hosts (Peterson et al., 2009), their role as selective agents on insect life histories must not be overlooked. However, studies approaching host–pathogen interactions from this perspective appear to be remarkably scarce, being mostly limited to work on viruses. Baculoviruses in particular are known to have substantial ecological impact on their hosts, including selective effects on habitat use by the insects (Kamita et al., 2005; Moreau & Lucarotti, 2007; Vega & Kaya, 2012).

Fungi constitute a significant group of insect pathogens (Lacey et al., 2015; Vega & Kaya, 2012). Entomopathogenicity in fungi has been shown to have evolved multiple times as a transition from saprotrophic feeding on decaying plant material, with the endophytic lifestyle as a likely intermediate state (Humber, 2008; St. Leger & Wang, 2020). Some entomopathogenic fungi, especially from the order Hypocreales, may continue to live as saprotrophs on the remnants of their dead hosts, persist dormant in soil, or occupy plant tissues as endophytes (Boomsma et al., 2014; Lacey et al., 2015; Samson et al., 2013). Consequently, the line between saprotrophs, endophytes, and entomopathogens is often difficult to draw. The emerging consensus is, however, that entomopathogenic fungi should be defined as those having evolved physiological and behavioral traits which allow them to penetrate the insects’ primary defense mechanism – the cuticle (Boomsma et al., 2014; St. Leger & Wang, 2020). Entomopathogens are ecologically diverse, being frequently classified into three categories. First, various fungi can be seen as facultative entomopathogens that use insects only in favorable conditions, otherwise leading the lives of saprotrophs or endophytes (St. Leger & Wang, 2020). Much better known, however, are the obligatory entomopathogens, representing either specialists – able to use just a small fraction of insect diversity – or generalists that infect insects from different clades.

The ecological role of entomopathogenic fungi is much less known than that of viruses, parasitoids, or vertebrate predators of insects. Our understanding of the ecology of insect–fungus interactions is still largely limited to basic observational research and studies aimed to unlock the potential of pathogenic insects as inundative biocontrol agents in agriculture (Elliot et al., 2000; Vega et al., 2009; Vega & Kaya, 2012). As only 12 fungal species are actively used in biocontrol, the remaining 750–1000 entomopathogenic species known so far (Faria & Wraight, 2007; Vega & Kaya, 2012; Wang & Wang, 2017) have largely escaped research attention beyond taxonomy. On the other hand, the bias toward agricultural systems has left little attention to the role of entomopathogenic fungi in studies on the ecology of wild insect populations, especially in the evolutionary context. Nevertheless, spatiotemporal patterns of fungal infections (see Barta & Cagáň, 2003; Filotas & Hajek, 2004; Lopez Lastra et al., 2006, for the fungi from the order Entomophthorales) have an undeniable potential to impose selective pressures on key ecological traits of insects, determining the patterns of where and when the life is safest. For example, infection risks which are variable in time should contribute to determining the optimal phenology (see Barta & Cagáň, 2006, for an example), i.e., the timing of the occurrence of different life stages in seasonal environments. On the other hand, especially because food plants can mediate insect–fungal pathogen relationships (Cory & Hoover, 2006; Lacey et al., 2015), the risk to catch a fungal disease may contribute to the evolution of host use in insect herbivores (Vega et al., 2009).

In the present study, we aim at evaluating the determinants of the risk of dying from a fungal infection in three lepidopteran species. Due to the obvious complications related to tracing the fate of insect individuals in nature, we chose a semi‐natural experimental design. In particular, the insects were reared from egg to adult in captive conditions in a natural habitat of the study species and fed with food plants of strictly local origin. Mortality caused by the fungi was recorded relative to food plant species, study site (the location where food plants were collected), phenological phase, and food limitation treatments used to manipulate the physiological condition of the insects. In addition to revealing the environmental determinants of the prevalence of fungal infections – being discussed in the context of evolutionary ecology of insects –, the present study serves the task of describing the still poorly known species composition of entomopathogenic fungi infecting natural populations of folivorous lepidopterans.

## 2 MATERIALS AND METHODS

### 2.1 Study system

The study was performed at a field station in Rõka village, Tartu County, Estonia (58°14′44″N, 27°17′54″E). The site is characterized by hemiboreal mixed forest stands with *Picea abies* and *Pinus sylvestris* as the dominant tree species.

Selection of insect species for the study was primarily based on their abundance at the study site and the authors’ previous experience with rearing them (Kivelä et al., 2020; Meister et al., 2017, 2018). Polyphagous species were preferred to facilitate studying the effects of different food plants. Additional selection criteria were overwintering in the pupal stage, and the females’ readiness to oviposit in captivity. Accordingly, three moth species were included in the study. In particular, *Acronicta rumicis* (Noctuidae: Acronictinae) is a polyphagous moth feeding on a wide variety of broadleaved woody and herbaceous plants. Pupation occurs aboveground between the parts of food plants or in leaf litter. The species is facultatively bivoltine (flying in June and August) in the study area. *Hypomecis atomaria* (Geometridae: Ennominae; previously *Ematurga atomaria*, see Murillo‐Ramos et al., 2021, for the recent nomenclatural change) is a polyphagous moth feeding on a wide variety of woody and herbaceous plants, the larvae pupate in the soil. *Cabera pusaria* (Geometridae: Ennominae) is an oligophagous moth feeding on trees mainly from the genera *Alnus* and *Betula*, similarly pupating in the soil. This species is bivoltine (flying in June and August) in the study area. All of the studied species are solitary external feeders on the leaves of their food plants, and all are medium sized with wingspan of roughly 3 cm. The larval period lasts for about 1.5 months, and consists of five instars (pers. observation).

Adult moths were collected in May and June 2019 in forest landscape at the distance of 0.5–2 km from the field station using netting, light trapping, and sugar baits. Collected females were placed into plastic vials accompanied with a leaf of larval food plant to induce oviposition (Tammaru & Javoiš, 2000). The leaf was removed after successful egg laying to exclude any uncontrolled feeding by the hatchlings.

The summer of 2019 was usual in terms of weather conditions, average daily temperatures in June, July, and August were 17.6°C, 16.4°C, and 16.6°C, respectively. Temperatures ranged from 25 to 30°C at daytime and 10–15°C during the nights. Average rainfall in June, July, and August was 49 mm, 68 mm, and 50 mm, respectively.

### 2.2 Study design

From June to September 2019, the larvae were reared individually in 50 ml plastic vials with pierced lids in outdoor conditions at the field station. Rearing vials were spatially randomized on rearing trays with respect to species and treatments (below). The trays were stored under a shade that protected them from direct sunlight. Sections of plants the larvae fed on were replaced every 3rd day. Date of pupation was recorded to facilitate calculation of the length of the development (= larval) period. Seven days after pupation, the pupae were weighted and put into vials with *Sphagnum* sp. to ensure stable humidity. *Sphagnum* is widely used as a “non‐infectious” substrate for lepidopteran pupae being known to harbor a mixture of antiseptic phenols (Drobnik & Stebel, 2017). Unlike most other organic substrates, it never develops an overgrowth of saprotrophic fungi which would obviously have been undesirable in the present study. *Sphagnum* moss was collected within 700 m from the field station. The pupae were overwintered in the study area, in a cellar next to the field station (100 m) at around 0–4°C from the end of September 2019 until the beginning of April 2020.

The experiment to study the determinants of the incidence of fungal infections was performed under a four‐factor (moth species, food plant, study site, and food limitation treatment) crossed design. Each individual larva was fed with the leaves of one particular plant species collected from a particular study site throughout its entire development; food limitation treatment was either applied or not. All the treatments were assigned randomly to individual larvae, paying attention to equal representation of broods (offspring of individual female) in the treatment groups. Last, considering the possibility that the incidence of fungal infections may change in the course of the season, we ensured that the timing of larval development period – formalized as hatching date of the larvae (June 7th until July 25th) – varied across the studied sample.

We focused on the effects of plant species because the food plant constitutes a major element of the environment for any herbivore in general, and specifically because of the possibility that some entomopathogenic fungi may also occur as endophytes (Rodriguez et al., 2009; Vega et al., 2009). Three forest plant species common at the study site were included into the study as hosts of the larvae: *Rubus idaeus* (Rosaceae), *Vaccinium myrtillus* (Ericaceae), and *Betula pubescens* (Betulaceae). As *B*. *pubescens* was not present at one of the study sites (below), it was replaced by the closely related *B*. *pendula*, and we thus refer to the birches as *Betula* sp. hereafter. Of the moths, *C*. *pusaria* was able to develop on *Betula* sp. only, so that the other two plants were not offered to the larvae of this species.

To reveal potential small‐scale spatial differences in the incidence of fungi, we collected the food plants from three distinct study sites (within the radius of 10 m); the sites were separated from each other (and the field station) by 500–1000 m of forest landscape (see map at DataCite https://doi.org/10.15156/BIO/2483897). At each of the three sites, plant leaves that the larvae were fed on were collected from two individual *Betula* trees; each larva was fed with the leaves of one individual tree throughout its development. Plant individuals could not be distinguished in the vegetatively propagating *R*. *idaeus* and *V*. *myrtillus*. For these species, the leaves were collected haphazardly from the study sites. We did not observe any signs of outbreaks of herbivorous insects in any of these sites, neither were the larvae of our study species encountered when foliage was collected for the experiment.

As physiological condition of the insect is likely to affect its susceptibility to infections (Murphy & Loewy, 2015), a food limitation treatment was imposed on half of the reared larvae throughout their last larval instar. Specifically, the food limitation treatment larvae were denied access to food plants every other day (24 h with food: 24 h without, see Tammaru et al., 2015, for methods) with the aim to reduce their growth rates and final weights.

### 2.3 Recording fungal infections

Throughout the development of the larvae, the insects were monitored for visually detectable signs of fungal infections with a 2‐ to 3‐day interval. After overwintering, in April to May 2020, the pupae were kept indoors at room temperature, and inspected daily for the incidence of fungi and emerged adults. Once a fungus was noted, the vial was kept closed and was transferred to the laboratory at the University of Tartu. Thereafter, the living fungus was isolated onto 2% malt extract agar (Oxoid, Cambridge, UK), supplemented with antibiotics (1% of streptomycin and tetracycline). Insects that died not showing any signs of fungal infections (in either larval or pupal stage), as well as those survived into the adult stage, were scored as not infected by a fungus. In no cases, visual signs of fungal infection were detected on a living insect.

### 2.4 Identification of fungi

After 1 to 3 weeks of growth, the fungal isolates (101 in total) were grouped into distinct morphotypes according to their characteristics studied using a stereo and/or dissecting microscope. Representatives of each morphotype (83 samples in total) were subjected to DNA barcoding. The procedures of growing mycelium, extracting DNA, conducting PCR, and sequencing followed the protocols described by Põldmaa et al. (2019). Species identification mostly relied on the assignment of the obtained ITS rDNA, the fungal DNA barcode marker, sequences to UNITE species hypotheses (SH; Kõljalg et al., 2020). The Basic Local Alignment Search Tool (BLASTn) at National Centre of Biotechnology Information (NCBI Resource Coordinators, 2016) was used to check for similar sequences not yet incorporated into the UNITE SHs. For distinguishing between closely related species (hypotheses) of Ascomycetes, the distance threshold <1% is usually chosen as was the case in our study (Table 1.), implying that each sequence in one SH must have a distance less than 1% to at least one sequence in that SH. The advantage of the SH approach is that regardless of the availability of a Latin binomial, unique persistent identifiers, assigned to all SHs in the form of DOIs, allow unambiguous communication about the identity of studied organisms. All specimens infected with a fungus were deposited at the fungarium (accession numbers TUF133197‐133317) and representative isolates at the microbial culture collection (TFC) of the Natural History Museum and Botanical Garden, University of Tartu. All sequences along with their metadata were uploaded to UNITE, using PlutoF, a data management and publishing platform (Abarenkov et al., 2010), and representatives of each species also to GenBank (accession numbers: OK649241‐OK649260). Information on collecting sites and dates, for all specimens deposited at TUF, as well as obtained sequences and images have been published at https://doi.org/10.15156/BIO/2483897.

**TABLE 1:** Fungal species detected on lepidopteran hosts in an outdoor rearing experiment

Order/family	Fungal species	SH	Host insects and cases detected
Cladosporiales/Cladosporiaceae	*Cladosporium herbarum* (Pers.) Link	SH1572792.08FU	*C*. *pusaria* 1 L *H*. *atomaria* 1 L
Eurotiales/Aspergillaceae	*Aspergillus versicolor* (Vuill.) Tirab.	SH1649133.08FU	*A*. *rumicis* 1 P
	*Penicillium chrysogenum* Thom	SH2189908.08FU	*A*. *rumicis* 1 P
	*Penicillium godlewskii* K.W. Zaleski	SH2189963.08FU	*A*. *rumicis* 1 P
	*Penicillium bialowiezense* K.W. Zaleski	SH2189921.08FU	*A*. *rumicis* 1 L *H*. *atomaria* 1 L
	*Penicillium polonicum* K.M. Zalessky	SH1529984.08FU	*C*. *pusaria* 2 L *H*. *atomaria* 1 L
	*Penicillium velutinum* J.F.H. Beyma	SH2189995.08FU	*A*. *rumicis* 1 L
	*Penicillium* sp. 1^a^	SH1537860.08FU	*A*. *rumicis* 1 L + 1 P *C*. *pusaria* 3 L *H*. *atomaria* 1 L
	*Penicillium* sp. 2^b^	SH2283940.08FU	*A*. *rumicis* 2 L + 1 P *C*. *pusaria* 6 L *H*. *atomaria* 1 L
Hypocreales/Cordycipitaceae	*Akanthomyces muscarius* (Petch) Spatafora, Kepler & B. Shrestha	SH1886969.08FU	*A*. *rumicis* 3 L + 4 P *C*. *pusaria* 4 L + 1 P *H*. *atomaria* 2 P
	*Beauveria pseudobassiana* S.A. Rehner & Humber	SH2173947.08FU	*A*. *rumicis* 1 L+ 1 P *C*. *pusaria* 2 L *H*. *atomaria* 4 P
	*Cordyceps militaris* (L.) Fr.	SH2173962.08FU	*C*. *pusaria* 1 P
	*Leptobacillium leptobactrum* (W. Gams) Zare & W. Gams	SH1529400.08FU	*A*. *rumicis* 1 P
	*Samsoniella* cf. *hepiali* (Q.T. Chen & R.Q. Dai ex R.Q. Dai, X.M. Li, A.J. Shao, Shu F. Lin, J.L. Lan, Wei H. Chen & C.Y. Shen) H. Yu, R.Q. Dai, Y.B. Wang, Y. Wang & Zhu L. Yang^c^	SH2173953.08FU	*A*. *rumicis* 1 L + 5 P *C*. *pusaria* 2 L + 3 P *H*. *atomaria* 23 P
	*Simplicillium filiforme* R.M.F. Silva, R.J.V. Oliveira, Souza‐Motta, J.L. Bezerra & G.A. Silva	SH1529405.08FU	*H*. *atomaria* 1 P
	*Simplicillium lamellicola* (F.E.V. Sm.) Zare & W. Gams	SH1584062.08FU	*A*. *rumicis* 2 P *C*. *pusaria* 1 L + 1 P *H*. *atomaria* 1 P
Hypocreaceae	*Trichoderma viride* Pers.	SH2303512.08FU	*H*. *atomaria* 1 P
Nectriaceae	*Fusarium tricinctum* species complex	SH2229701.08FU	*A*. *rumicis* 1 P *C*. *pusaria* 2 L
	*Fusarium solani* species complex	SH1546416.08FU	*A*. *rumicis* 1 P *H*. *atomaria* 1 P
	*Mariannaea camptospora* Samson	SH1506679.08FU	*H*. *atomaria* 1 P
Sarocladiaceae	*Sarocladium strictum* (W. Gams) Summerb.	SH1541921.08FU	*A*. *rumicis* 1 P
Mortierellales/Mortierellaceae	*Mortierella jenkinii* (A.L. Sm.) Naumov	SH1629839.08FU	*A*. *rumicis* 1 L
Pleosporales/Pleosporaceae	*Alternaria* sp.	SH1526648.08FU	*H*. *atomaria* 1 L

Note

For all fungal species, we present codes of UNITE species hypothesis (SH) to which the ITS rDNA sequences were assigned. Number of host insect individuals affected (by species and developmental stages) is indicated for each fungal species. L – on larvae, P – on pupae.

^a^ This SH consists mostly of isolates identified as *P*. *brevicompactum* Dierckx or *P*. *kongii* L. Wang, and one *P*. *patris*‐*mei* K.W. Zaleski which was isolated from fruiting body of the entomopathogen *Ophiocordyceps* sp. in India (GenBank MN744824). Almost identical sequences (e.g., GenBank MN636238) originate from a fungus identified as *P*. *brevicompactum* and isolated from *Varroa destructor* in Switzerland.

^b^ Six isolates, identified as *P*. *spinulosum* Thom, with ITS sequences identical to those obtained from the 10 isolates in this study, were isolated from *H*. *atomaria*, *C*. *pusaria*, and an unidentified lepidopteran pupae by the authors of this study (Gielen et al., 2021). The species identity yet needs to be resolved as this SH does not include any sequences from type material while originating from specimens identified as *P*. *spinulosum*, *P*. *glabrum* (Wehmer) Westling, and *P*. *thomii* Maire.

^c^ ITS sequences from our strains were identical to 174 sequences available at INSD, originating from various parts of the world, including the ex‐type sequence. On morphological basis, the species has been identified only from China and Vietnam (Wang et al., 2020) and the conspecificity of respective collections with our and other strains from geographically distant regions warrants further studies.

### 2.5 Statistical analysis

In the first set of the analyses, the incidence of fungal infection (as a binary trait: yes/no) was analyzed as dependent on moth species, food plant, study site, and food limitation treatment, with egg hatching date as an additional continuous variable. Generalized linear models were constructed for this purpose. Brood (offspring of an individual female) was initially included as a random variable, but dropped from the final models as no effects could be shown. The analysis was performed separately for infections detected in the larval and in the pupal stage, and for these two subsets combined. In addition, the data were analyzed separately by insect species; and separately by particular fungal taxa abundant enough for a meaningful analysis.

In the second set of the analyses, we compared species composition of the recorded assemblages of fungi as dependent on the affected life stage of the host (fungi recorded on larvae vs. pupae), and all the independent variables considered in the first set. Multinomial regression was applied. The frequencies of individual fungal species were too low to allow us to meaningfully characterize the assemblages at the level of individual species. Consequently, for the purposes of the analysis, several species were combined in one category. Accordingly, we subdivided the fungi into the following three categories: (1) Cordycipitaceae, (2) Aspergillaceae +Nectriaceae, and (3) all remaining taxa (see *Discussion*, for justification).

All statistics were done in R version 4.0.4 using packages lme4 (Bates et al., 2015) and car (Fox & Weisberg, 2018) for the first set of analyses, and nnet (Venables & Ripley, 2013) and car (Fox & Weisberg, 2018) for the second set.

## 3 RESULTS

**FIGURE 1:**
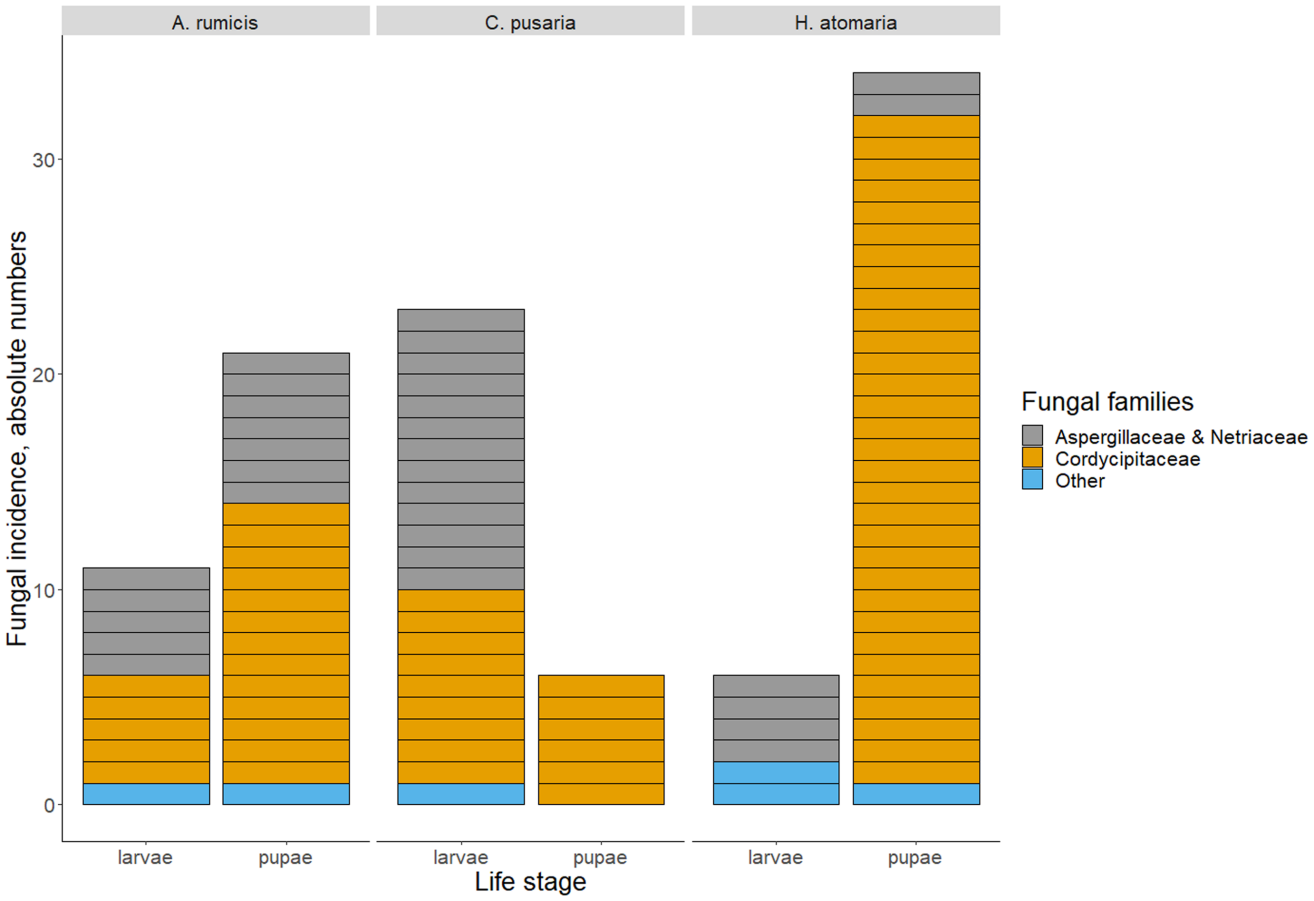
The number of cases in which a fungal pathogen was recorded, presented with respect to lepidopteran species (hosts), developmental stage of the host, and taxonomic affiliation of the fungus. AR, *Acronicta rumicis*; CP, *Cabera pusaria*; HA, *Hypomecis atomaria*

Summed over the three moth species, 1339 individuals entered the experiment as newly hatched larvae, and 565 of the insects survived to the adult stage. Fungal infections were detected in 101 individuals (7.5% of the total sample, or in 13% of those which died before the adult stage), 40 on larvae and 61 on pupae. Fungi were detected on 51.2%, 40%, and 73.9% dead pupae of *A*. *rumicis*, *C*. *pusaria*, and *H*. *atomaria*, respectively. Fungal infections were observed on both larvae and pupae, with the relative frequency of affected developmental stages differing among the host species (Chi‐square = 180.58, *p* < .001, Figure 1). As many as 23 species‐level taxa of fungi were detected on immature lepidopterans. These represented eight families from five orders (Table 1). All individual insect–fungus–host plant records and representative images of voucher material can be retrieved from https://doi.org/10.15156/BIO/2483897.

**FIGURE 2:**
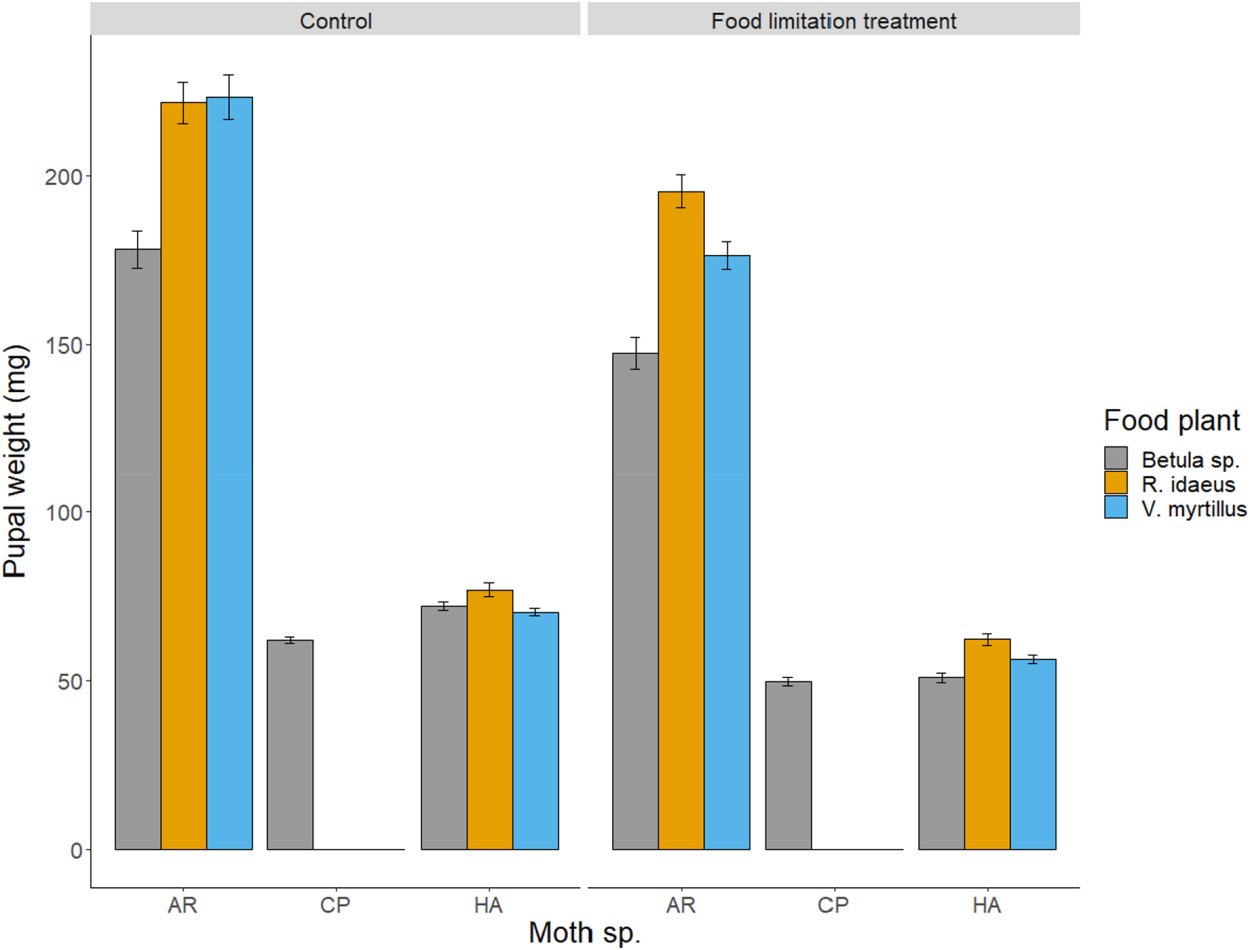
The effect of food limitation treatment (*F*_1, 661_ = 156.6, *p* < .001, in the total data set) and larval food plant (*F*_2, 661_ = 400.6, *p* < .001) on final body size in three lepidopteran species, mean ± SE. AR, *Acronicta rumicis*; CP, *Cabera pusaria*; HA, *Hypomecis atomaria*

The applied treatments were effective in creating variation in the developmental schedules of the moths: the larvae which were food limited in their last instar attained lower pupal weights and had longer development periods; in addition, the performance of insects differed among the food plants (Figure 2). There was also considerable phenological variance in *C*. *pusaria* and *H*. *atomaria* (SD of hatching 8.5 and 7.5 days, respectively); the first and last larvae in the sample hatched 41 (*C*. *pusaria*) and 25 (*H*. *atomaria*) days apart.

Overall, the effect of the predictor variables on the probability to gain fungal infection was relatively weak and inconsistent across the subsets of the data. In particular, entomopathogenic fungi occurred in all moth species, with qualitatively similar but still significantly different frequencies (Table 2, Figure 1), see Table 3 for statistics. All food plants, all study sites, and both food limitation treatments were associated with comparable frequencies of entomopathogenic fungi. In the analysis of the total data set, the only predictor attaining statistical significance was study site (Table 3): the probability to gain a fungus was 1.8 times higher at the most “infected” site compared to the least “infected” one. This difference was due to differential expression of fungi on larvae, but not pupae, and attained significance in *A*. *rumicis* only, with *C*. *pusaria* nevertheless showing a difference in the same direction (Table 3). The effect of food plant was apparent in just one subset of the data (two times higher infection probability of *A*. *rumicis* larvae on *Betula* sp.), which was also the case for phenology (larvae of *H*. *atomaria* hatched on Weeks 23–24 had three times higher risk of infection than larvae hatched on Weeks 26–27).

**TABLE 2:** Number of larvae reared, adults emerged, and mortality from fungal infections in different developmental stages in an experimental study on the determinants of the prevalence of entomopathogenic fungi in folivorous Lepidoptera

Insect species	Number of larvae hatched	Number of adults eclosed	Number of individuals with fungus	% of fungus mortality total	% of fungus mortality in larvae	% of fungus mortality in pupae
*A. rumicis*	391	173	32	8.2	2.8	5.4
*C. pusaria*	502	149	29	5.8	4.6	1.2
*H. atomaria*	446	243	40	9.0	1.3	7.6

**TABLE 3:** The incidence of fungal infections (as a binary trait: yes/no) as dependent on lepidopteran species (the host for the fungi; abbreviated as “Lep. sp.”), study site (food plant collection locality), food plant species, phenological phase (hatching date of the larvae) of the insect, and food limitation treatment as analyzed by generalized linear models, type III analysis

	Total			Larvae			Pupae		
	df	*χ* ^2^	*p*	df	*χ* ^2^	*p*	df	*χ* ^2^	*p*
Total data									
Lep. sp.	2	3.96	.14	**2**	**9.03**	.**011**	**2**	**10.23**	.**006**
Site	**2**	**6.99**	.**030**	**2**	**8.18**	.**017**	2	1.33	.52
Food (Lep. sp.)	4	5.07	.28	4	8.40	.078	4	4.97	.29
Hatching date (Lep. sp.)	3	4.84	.18	3	0.41	.94	3	2.45	.48
Treatment^a^							1	3.27	.07
Sample size	1339			1339			667		
*Acronicta rumicis*									
Site	**2**	**8.19**	.**017**	2	4.24	.12	2	2.99	.22
Food	2	3.57	.17	**2**	**7.72**	.**021**	2	4.07	.13
Hatching date	1	0.11	.74	1	0.43	.51	1	0.22	.64
Treatment							1	0.13	.72
Sample size	391			391			214		
*Cabera pusaria*									
Site	2	4.96	.08	**2**	**7.21**	.**027**	2	0.37	.83
Hatching date	1	0.92	.34	1	0.006	.94	1	1.36	.24
Treatment							1	1.09	.30
Sample size	502			502			164		
*Hypomecis atomaria*									
Site	2	0.33	.85	2	1.01	.60	2	0.19	.91
Food	2	1.52	.47	2	0.66	.72	2	0.82	.66
Hatching date	**1**	**4.09**	.**043**	1	0.001	.97	1	1.04	.31
Treatment							1	2.96	.09
Sample size	446			446			289		

Note

The effects of food plant and hatching date are nested within insect species. The analysis of the total data set is followed by analyses performed separately by particular insect species. Simplification of the models (omission of non‐significant effects) did not lead to any qualitatively different results.

Bold are those determinants that aquired statistical significance.

^a^ As the food limitation treatments were applied in the final larval instar, this factor cannot be considered when analyzing mortality which occurred prior to the pupal stage.

For Cordycipitaceae analyzed separately, the difference among moth species was more pronounced (Table 4, Figure 1), and food limitation treatment had a significant effect: unexpectedly, the food‐limited insects were less likely to gain a fungal infection in pupal stage (5.3% in food limited vs. 9.4% in control group). Two of the most numerous fungal species (Table 1.) were also analyzed separately. For *Samsoniella* cf. *hepiali*, there was higher incidence of this entomopathogenic fungus in *H*. *atomaria* than in the two other moth species (Tables 1 and 4). More importantly, the risk of becoming infected by *Akanthomyces muscarius* was strongly dependent on food plant (Table 4): actually, all 14 records originated from lepidopterans fed with *Betula* spp. (several records at all sites, and in all moth species, Table 1).

**TABLE 4:** Determinants of the incidence of fungal infections (as a binary trait: yes/no) separately by the most abundant fungal taxa. See Table 3 for further explanations

	Total mortality			Caterpillar mortality			Pupal mortality		
	df	*χ* ^2^	*p*	df	*χ* ^2^	*p*	df	*χ* ^2^	*p*
*Cordycipitaceae*									
Lep. sp.	**2**	**8.18**	.**017**	**2**	**11.80**	.**003**	**2**	**9.66**	.**008**
Site	2	1.98	.37	2	1.87	.39	2	1.22	.54
Food (Lep. sp.)	4	4.09	.39	4	3.34	.50	4	6.31	.18
Hatching date (Lep. sp.)	3	5.50	.14	3	1.25	.74	3	4.82	.19
Treatment							**1**	.**023**
Sample size	1339			1339			667		
*Akanthomyces muscarius*									
Lepidoptera sp.	2	3.79	.15	2	5.74	.057	2	2.25	.32
Site	2	1.39	.50	2	1.56	.46	2	1.08	.58
Food (Lep. sp.)	**4**	**12.63**	.**013**	4	4.24	.37	**4**	**11.78**	.**019**
Treatment							1	2.57	.11
Sample size	1339			1339			667		
*Samsoniella* cf. *hepiali*									
Lepidoptera sp.	**2**	**17.67**	**<.001**	2	2.57	.28	**2**	**13.65**	.**0011**
Food (Lep. sp.)	4	2.23	.69	4	1.39	.85	4	1.66	.80
Hatching date (Lep. sp.)	3	6.36	.10	3	0.45	.93	3	3.09	.38
Treatment							1	3.15	.075
Sample size	1339			1339			667		

Note

Bold are those determinants that aquired statistical significance.

Examining the magnitudes of the differences associated with significant effects (published at https://doi.org/10.15156/BIO/2483897) allowed us to conclude that the power of our analysis was sufficient to detect about two‐ (analyses of the total data set) to threefold (analyses by particular species) differences in the infection rate. Importantly, our conclusion about the limited influence of the studied ecological factors is primarily based on inconsistency of the effects across subsamples of the data, and not specifically on failure to attain statistical significance.

In the analyses of fungal communities, the three categories – (1) Cordycipitaceae, (2) Aspergillaceae + Nectriaceae, and (3) all remaining fungi – were unequally represented among larvae and pupae (Figure 1), with larvae having higher prevalence of fungi from the families Aspergillaceae and Nectriaceae and pupae being dominated by the family Cordycipitaceae. However, the assemblages of entomopathogenic fungi (as defined above) appeared not to differ among insect species, food plants, or study sites (Table 5).

**TABLE 5:** Determinants of fungal communities (three classes: Cordycipitaceae, Aspergillus & Nectriaceae, and other): results of a multinomial regression

Effect	df	*χ* ^2^	*p*
Developmental stage	**2**	**18.50**	**<.001**
Lepidoptera sp.	6	5.78	.45
Food (Lep. sp.)	16	9.50	.89
Start day (Lep. sp.)	8	8.51	.39

Note

Bold are those determinants that aquired statistical significance.

## 4 DISCUSSION

### 4.1 The assemblage of fungi infecting immature insects

The present study revealed high diversity of fungi infecting lepidopteran larvae in one particular location in the European hemiboreal forest zone. This conclusion is based on the results of a semi‐field experiment in which the larvae, even if kept in captive conditions, were reared on plant material of strictly local origin, and could thereby only be exposed to fungi representing local assemblages. Slight majority of fungi detected on dead insects (especially pupae) belonged to the family Cordycipitaceae, generally considered to comprise obligatory entomopathogens, with *Samsoniella* cf. *hepiali* as the numerically dominant species (Table 1). In terms of abundance, these fungi were followed by members of the family Aspergillaceae, as well as Nectriaceae, both of which include a large share of saprotrophs. Saprotrophic fungi may be detected on cadavers of insects which had died for other reasons, and may thus not have actually caused the death of the insects. Nevertheless, the ability of these fungi to invade living insects cannot by any means be excluded (Nicoletti & Becchimanzi, 2022; Poitevin et al., 2018; Sharma & Marques, 2018). For example, entomopathogenicity has been ascribed to several species and strains of *Fusarium*, including members of the *F*. *tricinctum* complex (Santos et al., 2020), one of which was detected also in this study. Moreover, according to our protocol, fungal infections were only recorded on larvae which had died between the inspection events, separated by 2 to 3 days. It appears unlikely (although not impossible) that a fungus could develop visually detectable structures within this time window if it had not been present in the still living larva. We thus treated all the recorded fungi as potentially having caused the deaths of the insects but we also focus on the obligatorily entomopathogenic Cordycipitaceae separately in our analyses.

The composition of fungal assemblages could not be shown to differ between the studied species of Lepidoptera (Figure 2, Table 5). In fact, all fungal species encountered more than twice were detected on more than one host species. This suggests that the studied fungi are not strict specialists in terms of host use. Indeed, as host populations are unevenly distributed in space and time, the fungi as organisms lacking the ability of active host search might not benefit from strong specialization (Meyling & Hajek, 2010). In contrast, fungal assemblages were found to differ considerably between the life stages of the insects (Figure 1). Larvae (rather than pupae) appeared to be more frequently infected by possibly facultative entomopathogens (i.e., other than Cordycipitaceae). It might well be the case that insect larvae constitute easier “prey” for the trophically less specialized Aspergillaceae and Nectriaceae, and their entomopathogenic qualities may have remained less known. Studies systematically comparing fungal pathogens of different life stages of particular insect species are needed to clarify this issue; such studies appear to be virtually lacking at the moment.

The present study had a focus on potential effects of larval food plant on the insect's probability of being infected. For this reason, to avoid soil‐borne infections, the larvae were allowed to pupate and were overwintered in *Sphagnum* moss (of local origin, though), known for its antiseptic properties. We thus assume that the fungi detected on moth pupae colonized the insects in the larval stage. As a drawback, we likely missed a component of relevant fungal biota (except for *A*. *rumicis*, which pupates aboveground) as the pupae did not come into contact with soil, frequently considered to be the main conidial storage space for entomopathogens (Chen et al., 2021; Samson et al., 2013). Therefore, our study may have failed to reveal, e.g., species of *Metarhizium*, a genus comprising some of the most well‐known and widespread entomopathogens. These fungi are isolated mainly from the rhizosphere (Kasambala Donga et al., 2021; Samson et al., 2013), although mainly from agricultural habitats (Chen et al., 2021).

### 4.2 Determinants of fungal prevalence

Overall, the prevalence of entomopathogenic fungi was not only moderate, invariably remaining below 10% across the subsets of the data (insect species, food plant species, and insect developmental stages) but also in no case was it equal to 0. This is consistent with some previously published observations (Barta & Cagáň, 2006; Gielen et al., 2021; Hall et al., 2012), although the reference points tend to be scarce: our study appears to be a rare example of an attempt to systematically investigate the prevalence of fungi in natural populations of insect herbivores. On the other hand, despite the systematic approach taken, it is obvious that the prevalences recorded in our semi‐natural setting may not quantitatively reflect corresponding values in nature. This is simply because our rearing conditions may have had both positive and negative effects on the development of the fungi in insect bodies. For this reason, we refrain from making far reaching conclusions from the absolute values of prevalence and focus on relationships among the variables which are less likely to be qualitatively affected by our experimental setting.

In general, the results of our manipulative study can be interpreted as providing evidence for relatively weak impact of the studied ecological factors on the probability to become infected and killed by a fungal pathogen. In the analysis of the total data set (all moth species), the effect of the site from which the food plants were collected was the only ecological factor attaining statistical significance, although caution is needed when interpreting this result as the site effect was largely limited to one subset (larval stage of one moth species). In our study, the insects were exposed to environmental conditions characteristic of the study sites only by mediation of food plants, which were collected at particular sites and used to feed the captive larvae. Quite clearly, conspecific plants growing at different sites may harbor different amounts of fungi for reasons unrelated to the properties of the plants, like a recent outbreak of a fungus at some site. Nevertheless, different plant individuals may host different amounts of fungi on their (leaf) surfaces as a result of microclimatic differences (Cory & Ericsson, 2010), or provide more or less favorable conditions for endophytes due to genetic or environmentally induced differences in plant biochemistry, physiology, and morphology (Cory & Ericsson, 2010; Elliot et al., 2000).

Somewhat unexpectedly, however, the effect of food plant species on the probability to be infected with a fungus was found in just one subset of the data. This may support the overall conclusion that the parameters of the food plant are not among the critical determinants of the risk of being killed by an entomopathogen. However, one fungal species *– Akanthomyces muscarius –* provided evidence of the opposite. In particular, all 14 cases when this fungus was recorded came from moths fed with *Betula* spp., representing all three study sites. Source information for DNA sequences accumulating in international nucleotide sequence databases suggests that several members of the genus *Akanthomyces* occur as endophytes (besides being recorded on arthropods). *Akanthomyces muscarius*, in particular, has been detected from a broad range of plant taxa, including trees (Nicoletti & Becchimanzi, 2020), in addition to its various animal hosts. Whether *A*. *muscarius* occurs as an endophyte in just a selection of food plant species of hemiboreal forest moths and whether the fungus–plant relationship can thereby have a role in shaping food plant use of these insects need further investigation. Thus far, however, members of the *A*. *attenuatus–lecanii–muscarius* complex, have not yet been found as endophytes in local trees (Küngas et al., 2020), including *Betula* spp. (Bahram et al., 2021) with very few records from soil samples in the area (Tedersoo et al., 2020).

Furthermore, the present study did not reveal any consistent effects of phenology (calendar date) on the probability to become infected. It is an intuitive expectation, supported by some evidence (Barta & Cagáň, 2006; Hall et al., 2012), that the abundance of pathogens should increase as the season progresses. Nevertheless, the only detected relationship was the opposite one, early hatched larvae of *H*. *atomaria* had a slightly higher risk of being infected by a fungus. This is despite the general tendency of deteriorating plant quality with date in temperate environments (Awmack & Leather, 2002) which should also affect the susceptibility of the herbivores to pathogens. Accordingly, the effect of manipulating the physiological condition of the larvae by a food limitation treatment had no consistent effect on the overall probability of becoming infected by a fungus. There was even a case of a weak effect in the opposite direction when only the obligatorily entomopathogenic Cordycipitaceae were considered. Lower probability of the food‐limited larvae to get infected might be related to their limited exposure to the inoculum carried by the food plants. However, as food‐limited larvae prolong their developmental periods in compensatory manner (Tammaru et al., 2015), we cannot expect this effect to be particularly strong.

Summing up the results of the present study did not provide strong evidence of an insect's risk of succumbing to a pathogenic fungus being variable enough in space or time to create substantial selective pressures on habitat or host preference or phenology. Somewhat unexpectedly, our study rather suggests that, in herbivorous insects, catching a fungal infection is largely a matter of chance; the incidence of pathogenic fungi was poorly predictable by the values of those environmental parameters, which are generally considered key determinants of the performance of herbivorous insects. This is consistent with the suggestion that the propagules of the fungi infecting folivorous insects are primarily air borne (Hesketh et al., 2010). However, the detected exclusive association of *A*. *muscarius* with feeding on *Betula* provides a convincing opposite example, and encourages further investigation. This case refers to the possibility of endophytic origin of lethal fungal infections on insects for which thus far no evidence has been presented, despite several reports on the effect of entomopathogenic endophytes on the performance of symptomless pests (Vidal & Jaber, 2015).

It can be speculated that both species richness and generalism of the entomopathogenic fungi contribute to the evenness and apparent randomness of fungus‐caused mortality in insects. In particular, ecological theory predicts that generalism leads to stability in ecological interactions. In contrast, in species‐poor communities of highly specialized species, we can primarily expect to see patterns in space and time (Begon et al., 2006; Price et al., 2011). Naturally, the results of a single case study should be treated with appropriate caution to avoid overinterpretation, but we believe that we have shown that experimental studies on the role of fungal pathogens in natural insect communities are feasible, and have the potential to deliver promising insights.

## AUTHOR CONTRIBUTIONS

**Robin Gielen:** Conceptualization (equal); Data curation (lead); Formal analysis (equal); Investigation (lead); Methodology (supporting); Writing – original draft (equal); Writing – review & editing (equal). **Kadri Põldmaa:** Conceptualization (equal); Data curation (lead); Funding acquisition (supporting); Investigation (equal); Methodology (equal); Supervision (supporting); Writing – original draft (equal); Writing – review & editing (equal). **Toomas Tammaru:** Conceptualization (equal); Data curation (supporting); Formal analysis (equal); Funding acquisition (lead); Investigation (supporting); Methodology (lead); Project administration (lead); Supervision (lead); Writing – original draft (equal); Writing – review & editing (equal).

## Data Availability

Metadata for all fungi detected in this study have been uploaded to PlutoF and can, along with DNA sequences and images, be accessed at https://doi.org/10.15156/BIO/2483897. All sequences are available at UNITE database (https://unite.ut.ee/; accessions UDB0780737–UDB0780774, UDB0802794–800, UDB01004393–396) and representative sequences also at GenBank (OK649241–OK649260).
